# Learning to Classify Organic and Conventional Wheat – A Machine Learning Driven Approach Using the MeltDB 2.0 Metabolomics Analysis Platform

**DOI:** 10.3389/fbioe.2015.00035

**Published:** 2015-03-24

**Authors:** Nikolas Kessler, Anja Bonte, Stefan P. Albaum, Paul Mäder, Monika Messmer, Alexander Goesmann, Karsten Niehaus, Georg Langenkämper, Tim W. Nattkemper

**Affiliations:** ^1^Biodata Mining Group, Faculty of Technology, Bielefeld University, Bielefeld, Germany; ^2^Bioinformatics Resource Facility, Center for Biotechnology, Bielefeld University, Bielefeld, Germany; ^3^Department of Safety and Quality of Cereals, Max Rubner-Institut, Detmold, Germany; ^4^Department of Soil Sciences, Research Institute of Organic Agriculture (FiBL), Frick, Switzerland; ^5^Department of Crop Sciences, Research Institute of Organic Agriculture (FiBL), Frick, Switzerland; ^6^Bioinformatics and Systems Biology, Justus-Liebig-University Gießen, Gießen, Germany; ^7^Department of Proteome and Metabolome Research, Faculty of Biology, Center for Biotechnology, Bielefeld University, Bielefeld, Germany

**Keywords:** metabolome informatics, statistics, metabolomics, computational metabolomics, organic farming, food authentication, machine learning

## Abstract

We present results of our machine learning approach to the problem of classifying GC-MS data originating from wheat grains of different farming systems. The aim is to investigate the potential of learning algorithms to classify GC-MS data to be either from conventionally grown or from organically grown samples and considering different cultivars. The motivation of our work is rather obvious nowadays: increased demand for organic food in post-industrialized societies and the necessity to prove organic food authenticity. The background of our data set is given by up to 11 wheat cultivars that have been cultivated in both farming systems, organic and conventional, throughout 3 years. More than 300 GC-MS measurements were recorded and subsequently processed and analyzed in the MeltDB 2.0 metabolomics analysis platform, being briefly outlined in this paper. We further describe how unsupervised (t-SNE, PCA) and supervised (SVM) methods can be applied for sample visualization and classification. Our results clearly show that years have most and wheat cultivars have second-most influence on the metabolic composition of a sample. We can also show that for a given year and cultivar, organic and conventional cultivation can be distinguished by machine-learning algorithms.

## 1 Introduction

The increasing awareness of the benefits of healthy eating has tremendously risen the popularity of organic food – a development that was not least stirred up by the manifold food scandals grabbing the headlines in recent years. Directly resulting from this popularity but in particular from organic food’s great market potential, there emerged a significant interest in the authenticity of food declared as organic (Capuano et al., 2013). Metabolomics technologies have proven successful for several task of food authentication (Cubero-Leon et al., 2014). In this study, we investigate the potential of metabolomics profiling techniques, bioinformatics, and machine learning to distinguish organically grown wheat from conventionally grown wheat. To this end, a total of more than 300 gas chromatography-mass spectrometry (GC-MS) measurements from both types of treatments were recorded and analyzed. Samples comprised 11 different wheat cultivars from up to 3 different years, obtained from the DOK field trial in Switzerland (Mäder et al., 2002). This comprehensive field trial compared organic and conventional farming systems, using strictly controlled conditions. In previous work (Bonte et al., 2014), we already presented metabolite profiling data obtained from the DOK wheat samples of the harvest year 2007. Röhlig and Engel (2010) have applied principal component analysis (PCA) and analysis of variance (ANOVA) to a very similar dataset. In the scope of this work, we substantially extended the DOK data basis from 2007 by additionally analyzing samples from the 2009 and 2010 harvest years. The particular focus of this work was placed on the potential of machine learning methods as tools for automated data classification. Furthermore, the new approach is metabolite-agnostic: it does not rely on correct metabolite identification and it does not rely on single biomarkers with significant level differences. The latter is a core advantage of this approach, as literature reveals that only slight (not significant) metabolite level changes can be accounted on the farming systems (Röhlig and Engel, 2010; Laursen et al., 2011; Bonte et al., 2014).

All GC-MS measurements were automatically preprocessed and then carefully annotated in our MeltDB 2.0 metabolomics analysis platform (Neuweger et al., 2008; Kessler et al., 2013). MeltDB allowed us to apply a well-established routine in high-dimensional molecular data analysis. After preprocessig (peak picking, normalization, profiling, etc) the data is represented as a table of dimension *n* × *D*, with *n* = number of samples and *D* = signal dimension (i.e., the metabolic profile). The first aim is to search for hidden regularities, relationships, and correlations in the data. To this end, unsupervised learning, i.e., dimensional reduction can be applied. Concretely, the two unsupervised methods, principal component analysis (PCA) and t-distributed stochastic neighbor embedding [t-SNE, (van der Maaten and Hinton, 2008)], were used to investigate the inter- and intra-class variances in the entire dataset as well as in particular subsets of the data.

Second, the data was analyzed toward the question, if it can be classified into distinct semantic categories (like conventional/organic treatment in this case). We, therefore, applied the two supervised machine learning methods random forests [RF, (Breiman, 2001)] as well as support vector machines [SVM, (Vapnik, 1999)]. The overall aim was to establish a classifier to distinguish between organic and conventional wheat, despite the influences of the years of growth and different cultivars.

In the following, the analytical approach is described in detail, as well as the separation results for the investigated factors treatment, cultivar, and year. All presented computational methods were implemented within the MeltDB 2.0 platform and can be applied on other datasets as well.

## 2 Materials and Methods

### 2.1 Plant material

Wheat grains of up to 11 different cultivars originated from the DOK (D: bio-dynamic, O: bio-organic, K: “konventionell” German for conventional, i.e., integrated, farming system) field trial, which is located at Therwil (7°33’ E, 47°30’ N) close to Basel (Switzerland). Detailed information on the DOK long-term field trail is given by Mäder et al. (2002). Wheat grains of the cultivar Runal were analyzed from the 3 harvest years, 2007, 2009, and 2010. In 2008, wheat was not grown in the trial. Further, the 10 wheat cultivars “Rouge de Bordeaux,” “Mont Calme 245,” “Probus,” “CCP” (composite cross-population; for ease of reading CCP is referred to as a cultivar), “Scaro,” “Sandomir,” “DJ 9714,” “Antonius,” “Caphorn,” and “Titlis” were integrated into the wheat plots of the long term trial of the harvest year 2007. In the 2010 cultivation period, cultivars “Mont Calme 245” and “DJ9714” were not available, leaving the remaining 8 cultivars mentioned previously for analysis in this work. A detailed description of the layout and design of the experiment comprising all winter wheat cultivars was published (Hildermann et al., 2009).

Thus only some essential information about the DOK field trial is considered here. The trial comprises several organic and conventional farming systems, each system being repeated in four field plots. The experimental design was a split plot with systems as the main factor and wheat cultivars as the secondary factor. For this work, we choose to analyze the two farming systems, biodynamic 2 (D), (henceforth, organic) and conventional (M). These two farming systems were quite different with respect to fertilization and further plant treatment (see below), but at the same time, were still within the range of standard organic and conventional farming.

The organic system received composted manure and slurry at a fertilization level of 1.4 livestock units per hectare, equivalent to 66 kg N(total) ha^−1^. Fertilization in the conventional system was done exclusively with mineral fertilizerat 140 kg N(total) ha^−1^. Both farming systems also differed in plant protection practice. The conventional system followed the guidelines of integrated farming, using fungicides, insecticides, and herbicides only if needed. The biodynamic farming allowed only mechanical plant treatments and indirect methods to control weeds, pests, and diseases. Grains of both farming systems were harvested when completely ripe, with moisture content below 140 g kg^−1^. Of each of the four individual field plots per agricultural system, one sample was taken for each cultivar and farming system. Before further experimental usage, grain material was stored at a constant temperature of 18°C.

### 2.2 Wheat sample preparation and GC-MS analysis

Cleaning of wheat samples from impurities and broken grains, grain storage, grinding and extraction as well as measurement of metabolites using GC-MS analysis was exactly performed as described by Bonte et al. (2014).

### 2.3 Data processing in MeltDB 2.0

All data gained by GC-MS analysis were preprocessed and annotated within the MeltDB 2.0 metabolomics software platform. Peaks were obtained using the Warped Peak Detection tool. Retention indices were obtained semi-automatically, using MeltDB’s RISimple tool and a manually defined list of expected retention times for each batch of measurements. Next, a profiling was run to annotate peaks that are common throughout multiple chromatograms, i.e., they have a similar retention index and a similar EIC spectrum. Similarly, all chromatograms were matched against reference spectra to annotate peaks as identified compounds were possible. However, the subsequent approach does not rely on the identification of compounds, but rather uses it to limit the feature space to molecules of potential biological interest. The parameterizations for these processing tools can be found in Table 1.

**Table 1:** **Parameters that were applied for preprocessing tools**.

Tool	Description	Parameter	Value
Warped peak detection	Mexican-wavelet based peak detection, which can be rerun locally (at certain RT).	FWHM	7
		SN	10
RISimple	Detects and tags retention indices based on heir characteristic spectra.	Ion filter	57, 71, 85, 99
Multiple profiling	Gives peaks across chromatograms a common TAG if they are similar.	Retention time window	20–35 s
Reference list	Annotates peaks that match reference spectra, uses dot-product.	RT Window	20 s

Results from automated metabolite identification were revised manually to discard erroneous annotations, but also to manually create annotations that were missed in a few chromatograms only. Peaks that were missed in a minority of samples only, were requantified using the Warped Peak Detection tool. Subsequently all data in the obtained feature table was centered and scaled using R.

In total, 313 samples were analyzed. From the years 2007, 2009, and 2010 these comprise 160, 16, and 137 samples, respectively. How many cultivars were available in each year is mentioned in Section 2.1. For each combination of year and cultivar, 13–16 samples comprising duplicate metabolite extractions for grains from most field plots were analyzed. From these, one half was treated organically, and the other half was treated conventionally. A detailed listing of all samples is given in Table 2.

**Table 2:** **Number of samples for each combination of factors “farming system,” “year,” and “cultivar”**.

Farming system	Year	Cultivar											
		Antonius	Caphorn	CCP	DJ 9714	Mont Calme 245	Probus	Rouge de Bordeaux	Runal	Sandomir	Scaro	Titlis	Σ
Conventional organic	2007	7	8	7	7	8	7	7	8	6	6	8	160
		8	7	7	7	7	8	8	7	8	7	7	
Conventional organic	2009								8				16
									8				
Conventional organic	2010	8	8	7			8	8	7	8	7	8	137
		8	8	8			8	8	7	7	7	7	

### 2.4 Unsupervised learning/dimensional reduction

The result of the preprocessing step is an *n* × *D* data table (*n* = 313, total number of samples (years, cultivars and treatments combined); *D* = 36, number of compounds consistently annotated in all samples). Additionally, subsets of this data table, for example, all samples from only 1 year or cultivar, have been analyzed too. Although it may be tempting to instantly apply supervised learning to the problem of classifying the data rows into conventional and biological treatment, we first applied some information visualization in advance to avoid unpleasant black box effects and to gain a mental model of the data. Information visualization uses different data displays which are inspected by human experts to understand the data or to build hypotheses for the hidden structures in the data. These are the foundation for any subsequent attempt to apply supervised learning. We propose to inspect displays obtained with two different dimensional reduction techniques. First, we applied PCA since this is a well-established statistics tool in high dimensional data analytics and is fully sufficient to understand data with a linear substructure. Since data stemming from systems biology experiments can not be expected to have such intrinsic linear structure, we used another method which has been proposed in the field of machine learning, the t-SNE. In several real world applications for computational biology (Jamieson et al., 2010; Bushati et al., 2011; Abdelmoula et al., 2014), t-SNE has shown to be capable of projecting non-linear data structure while well preserving the local features (i.e., neighborhoods) of the data.

The dimensional reductions were performed using the R statistical software (R Development Core Team, 2011) and the “tsne” package by Donaldson (2012).

### 2.5 Supervised learning/classification

The same *n* × *D* data table was used to explore whether a machine learning algorithm such as the Support Vector Machine [SVM, (Vapnik, 1999)] with a polynomial kernel (Karatzoglou et al., 2004) can be trained to classify the data rows into conventional and biological treatment. In a first step for each subset (e.g., data from 1 year only), the machine learning algorithm was trained and tested on 80% of the data (randomly selected). Afterwards, the remaining 20% out-of-the-bag data was used for validation, i.e., to finally evaluate the performance of the classifier construced using the 80% of the data. To train and optimize the SVM, a parameter tuning was performed using a 25-fold resampling for Leave-Group-Out-Cross-Validation (LGOCV) of the training partition. For this cross validation, again 75% of the training partition were used for training, and 25% were used for validation in each iteration. The best set of parameters, that led to the best accuracy according tothe LGOCV, was then once more validated on the 20% of the data that was kept back initially. The classification results on these latter 20% were evaluated in a confusion matrix to infer the accuracy of the trained SVM.

Using the very same subsets and partitions random forest [RF, (Liaw and Wiener, 2002)] were performed as well. The RF training was done with a 20-fold resampling and a parameter tune length of 12.

Supervised machine learning methods were performed in R as well, using the “caret” package (Kuhn, 2008; Kuhn et al., 2008).

## 3 Results

### 3.1 Unsupervised learning/dimensional reduction

**Figure 1:**
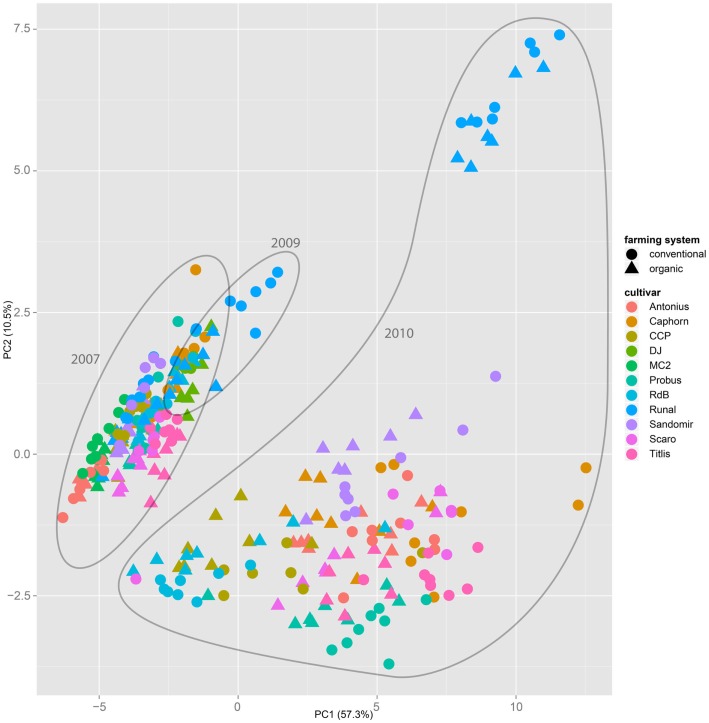
**The principal component analysis on the entire dataset of all samples throughout all years, cultivars, and treatments show that the first two components mainly separate samples by the factor year**. A separation by the factor farming system is not possible.

**Figure 2:**
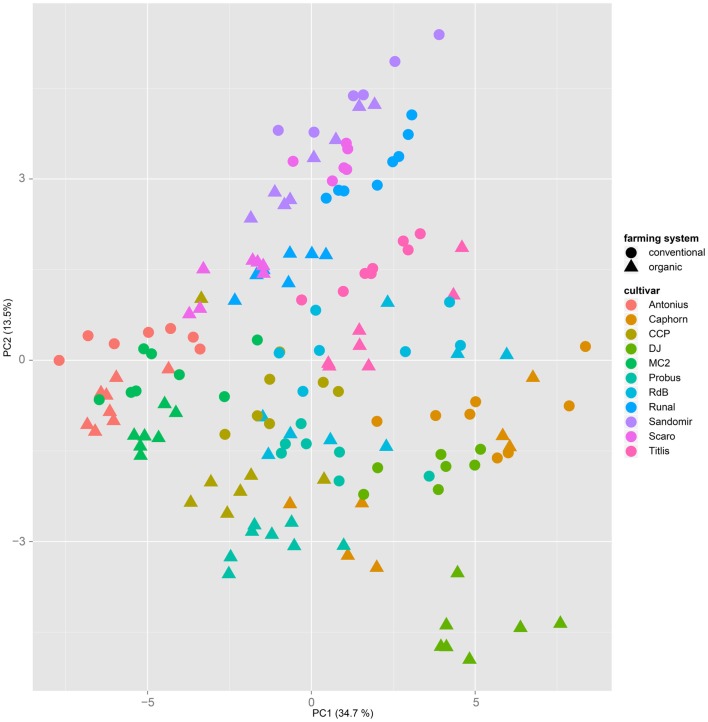
**A principal component analysis performed on a dataset from 1 year only will mainly cluster samples by their cultivar, regardless of the applied farming system**. This PCA is based on samples from the year 2007.

**Figure 3:**
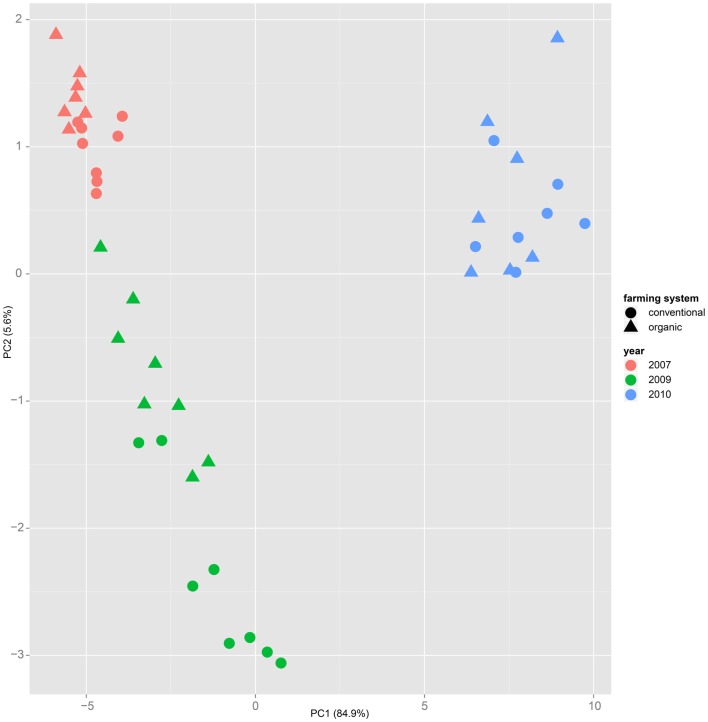
**Similar to Figure 1, in the principal component analysis on a dataset of only one cultivar – here “Runal” is shown – the first principal components separate samples by factor year**.

**Figure 4:**
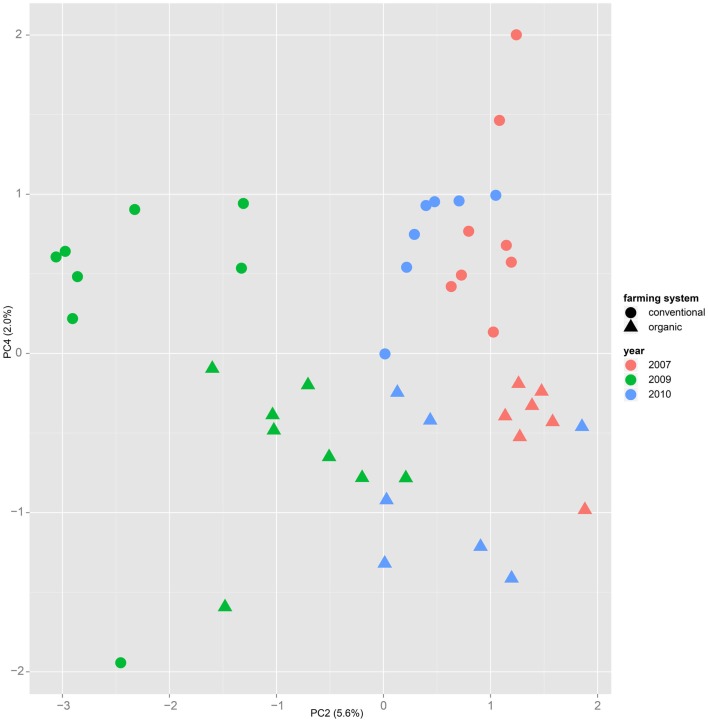
**Plotting samples from one cultivar (here “Runal”) along the principal components two and four show that a separation by farming system might be possible, even though the main variance is caused by the factor year**.

**Figure 5:**
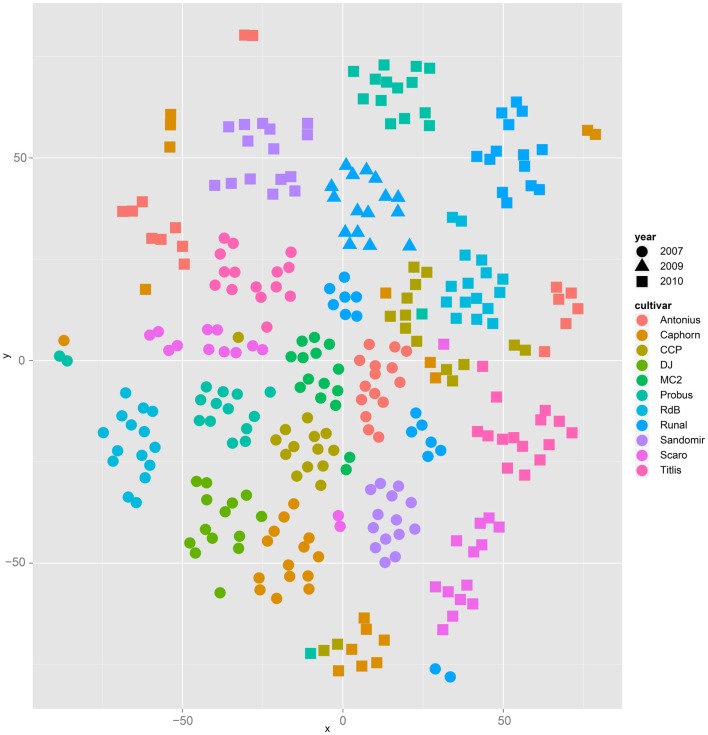
**The t-SNE method applied to all samples results in clusters and sub clusters formed according to the factor year and cultivar, respectively**.

**Figure 6:**
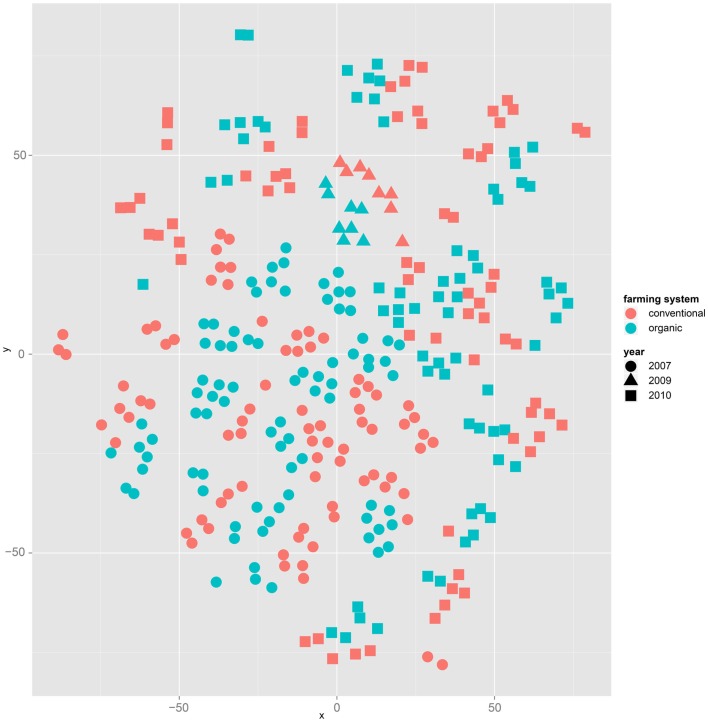
**The same t-SNE result as in Figure 5, but colored by farming system: clusters representing cultivars form subclusters according to the factor farming system**.

Both, PCA (in the first two principal components, see Figure 1) and t-SNE, are capable of separating the presented wheat samples into clusters according to the factor year. Within 1 year, the PCA will group samples according to cultivars, though with considerable overlap as can be seen in Figure 2. Conversely, within one cultivar samples will be grouped according to the year (see Figure 3). When 1 year and one cultivar are investigated in any combination, all data typically clusters into the two groups representing either dynamically or conventionally grown wheat. Most of these clusters show at least some overlap though. Figure 4 plots the second and fourth principal components of the PCA that has already been introduced in Figure 3. In this particular case, it is clearly visualized how the fourth principal component can be used to separate the samples according to the levels organic and conventional. The t-SNE method is less applicable to smaller datasets and thus was applied to the complete data table only. Figure 5 shows how t-SNE groups all samples by year at first, and then into subclusters according to their cultivars. The latter subclusters themselves are again split in two groups each, which correspond to the two farming systems, as can be seen in Figure 6. Figures 5 and 6 again visualize strikingly how the metabolic profile is mainly influenced by year, then by cultivar, and at least by the farming systems.

Nevertheless it is still obvious that the treatment caused measurable differences in the metabolic composition of the wheat samples. The first two principal components of the PCA in Figure 3 already reveal that the clusters for the years 2007, 2009, and 2010 are split in themselves to form subclusters of conventionally and organically grown wheat. This points out that later principal components with different loadings may expose structures in the data that are mainly based on the factor treatment.

### 3.2 Supervised learning/classification

The results from the PCAs revealed that there are structures in the data that allow for a separation of conventionally and organically grown wheat. Even though the main clustering is driven by factor year, these clusters still form subclusters according to cultivar, which again are clustered by the two farming systems. These substructures suggest that SVMs can be constructed to win classifiers for the problem. In fact, SVMs trained and tested on the entire dataset (all years, all cultivars, both treatments) to classify by treatment reached an accuracy of 0.9032 (*p*-value = 1.486e–11, see Table 3) on the validation set. Even better accuracies can be observed when investigating subsets of the data (for example, accuracy = 0.9677, *p*-value = 3.746e–08 within year 2007). But the smaller the subsets, the smaller the testing partitions, the less representative are any outcomes. Thus we will not trust the classifiers for in-cultivar or even in-year-and-cultivar problems to be flawless, even though in these cases accuracy values may approximate one easily.

**Table 3:** **Results of the support vector machines, trained and tested on different subsets of all samples**.

Trained on	Tested on	*n _Test_*	Accuracy	NIR^a^	*p*-Value^b^	Sensitivity	Specificity	PPV^c^	NPV^d^
2007	2007	31	0.9677	0.52	3.75e–08	1	0.9375	0.9375	1
2010	2010	26	0.8846	0.5	4.40e–05	0.9231	0.8462	0.8571	0.9167
2007	2010	137	0.5547	0.5	0.1333	0.2754	0.8382	0.6333	0.5327
2010	2007	160	0.5562	0.51	0.1177	0.8101	0.3086	0.5333	0.625
2007, 2009, 2010	2007, 2009, 2010	62	0.9032	0.5	1.49e–11	0.9032	0.9032	0.9032	0.9032

*Measures are given for the evaluation results and are based on the confusion matrix for classification as biological or conventional farming system*.

*^a^No information rate: the larger class percentage*.

*^b^Exact binomial test [accuracy > NIR]*.

*^c^Positive predictive value*.

*^d^Negative predictive value*.

The interesting question would be, if it is possible to obtain such a trained classifier from a number of (past) years that can then be applied to classify samples from another (e.g., the present) year. This, however, turns out to be not possible on the basis of the available data from the three growing seasons. For example, when a SVM, trained on data from 2007, is applied to classify data from 2010 it performs with an accuracy of 0.5547, which is hardly favorable to plain guesses. The reason for this poor performance seems to be the massive influence of the seasonal conditions, i.e., the factor “year.” This calls for continuing research using more samples from more years and cultivars to cover the molecular variance more appropriately. Estimations on the variable importance (Kuhn et al., 2008) for the 3 years were calculated based on the SVM results and added to the supplemental information. Here it is striking that e.g., *myo*-inositol, which has previously been reported as a potential marker for farming systems (Röhlig and Engel, 2010; Bonte et al., 2014), was most important for classification in 2007 but almost least important in 2009 and 2010. Such inhomogeneous variable importances additionally suggest a year-by-year strategy for training and classification.

Table 3 summarizes the SVM results. Please note that classification results for year 2009 are not reported here: with only one cultivar (Runal) and thus only 16 samples, the subset is too small to generate reliable results. The 2009 samples are part of the analysis of the entire dataset, though.

Overall random forest (RF) as described in the methods section led to similar classification results, but showed slightly lower accuracies in the in-year subsets. Thus no detailed results are shown in the manuscript. However, we explicitly do not suggest to ignore random forest as a potential alternative for support vector machines in this scope.

## 4 Discussion

The main goal of this study was to investigate whether a classification of organically and conventionally grown wheat can be done, based on GC-MS metabolite measurements of wheat grains from different years and cultivars. Results from the unsupervised machine learning methods PCA and t-SNE show that the strongest variation in the data can be found in samples from different years. This may be, in part, due to different environmental influences and also due to systematic errors that inevitably will occur in analyses from different years. Other studies report on the same obstructive effects (Röhlig and Engel, 2010; Laursen et al., 2011). On the other hand though, this allows to extend the data basis every year. This demands robust classifiers that are able to cope with these kinds of problems, besides “distracting” factors like year and cultivar. Further studies will additionally have to consider geographical influences on the metabolic composition of wheat grains.

Peaks from all 313 samples have been carefully annotated to achieve 36 consistently quantified features throughout the entire data set. These have first been explored with dimensional reduction methods like PCA and t-SNE to find the predominant structures in the data table. Then, supervised machine learning methods have been trained and applied to investigate in how far classifiers for organically and conventionally grown wheat can be created.

The considerably strong differences in samples from different years make it impossible though, to apply a classifier that was trained using data from year *a*_1_ to distinguish data from another year *a*_2_. To create a classifier for any year *a_x_*, data from this *a_x_* must be part of the training data set. PC analyses also suggest that it will be benefical to concentrate on one cultivar or to have a broad data basis of many cultivars to cover variances that derive from this factor.

Support vector machines trained and applied on all samples from the same year, as well as SVMs trained and tested on all years, performed with high accuracies above or close to 0.9. This clearly outperforms the ability of PCA to separate samples according to the applied farming system, unless samples derive from the same cultivar. For comparison, we also performed a study using Random Forests (Breiman, 2001) instead of SVMs for classification. Random Forests (RF) have the advantage to be much faster and more efficient than SVMs and they have the potential to offer some insight into the semantics of the decision function, but the parameters are more difficult to optimize. However, the classification performances were only slightly different from those obtained with SVMs and inferior in in-year analyses, so we did not include those in the manuscript.

The here presented machine learning tools are not meant to substitute traditional statistical methods, such as ANOVA, but provide a metabolite-agnostic approach for sample classification where reliable biomarkers are not known. Additionally, they may contribute a starting point for focused statistical analyses of single compounds that appear promising according to the computed variable importance estimations.

An analytical approach that aims more for specific compounds as biological markers can be found in the publication of Bonte et al. (2014), where more traditional statistical methods have been applied. The methods presented in the manuscript at hand do not depend on the identification of compounds or the determination of the biological meaning of any features. The approach rather relies on a consistently annotated data set. Nevertheless, it is constructive to do compound identification to be able to base further biomarker research on these studies. Additionally, reducing the feature set to verified biological compounds minimizes the risk of systematic errors through background noise. Variable importance estimations based on the SVM results of the 3 years have thus been added to the supplemental information. The integration of the discussed approaches might finally lead to a set of metabolites that can be used as reliable biomarkers for conventional or biodynamic farming systems.

## Author Contributions

Design and implementation of the DOK experiment: PM, MM. Conception and design of wet-lab experiments: AB, KN, GL. Conception and design of data analysis: NK, SA, AG, KN, TN. GC-MS measurements: AB. Data processing: NK, AB. Data analysis and software implementation: NK. Contribution of raw data/materials/analysis tools: SA, PM, MM, AG, KN, GL, TN. Preparation of the manuscript: NK, AB, SA, PM, MM, AG, KN, GL, TN.

## Conflict of Interest Statement

The authors declare that the research was conducted in the absence of any commercial or financial relationships that could be construed as a potential conflict of interest.

## Supplementary Material

The Supplementary Material for this article can be found online at http://journal.frontiersin.org/article/10.3389/fbioe.2015.00035/abstract
