# Supplementary material for: Learning to Classify Organic and Conventional Wheat – A Machine Learning Driven Approach Using the MeltDB 2.0 Metabolomics Analysis Platform
Source: Front Bioeng Biotechnol. 2015 Mar 24;3:35. doi: 10.3389/fbioe.2015.00035 (PMC4371749; doi:10.3389/fbioe.2015.00035)
Supplement: Supplementary file 1 [file Presentation_1.PDF]

Supplementary Material: Learning to classify organic and conventional wheat - a machine-learning driven approach using the MeltDB 2.0 metabolomics analysis platform

Nikolas Kessler [1,2], Anja Bonte [3], Stefan P. Albaum [2], Paul Mäder [4], Monika Messmer [5], Alexander Goesmann [6], Karsten Niehaus [7], Georg Langenkämper [3], and Tim W. Nattkemper [1*]

[1] *Biodata Mining Group, Faculty of Technology, Bielefeld University, Bielefeld, Germany*
[2] *Bioinformatics Resource Facility, Center for Biotechnology, Bielefeld University, Bielefeld, Germany*
[3] *Department of Safety and Quality of Cereals, Max Rubner-Institut, Detmold, Germany*
[4] *Department of Soil Sciences, Research Institute of Organic Agriculture (FiBL), Frick, Switzerland*
[5] *Department of Crop Sciences, Research Institute of Organic Agriculture (FiBL), Frick, Switzerland*
[6] *Bioinformatics and Systems Biology, Justus-Liebig-University Gießen, Gießen, Germany*
[7] *Department of Proteome and Metabolome Research, Center for Biotechnology, Faculty of Biology, Bielefeld University, Bielefeld, Germany*

Correspondence*:
Tim W. Nattkemper
Biodata Mining and Applied Neuroinformatics Group, Faculty of Technology, Bielefeld University, Universitätsstr. 25, 33615 Bielefeld, Germany,
tim.nattkemper@uni-bielefeld.de

1 DATA ANALYSIS EXAMPLE

The following code is an example of how the statistical analyses in the manuscript were generated. It is arranged in the style of a tutorial with explanatory comments inline. As data basis please find the txt file that is provided with the supplementary information. This file contains the signal abundances as exported from MeltDB. For simplicity please also load the two provided .R files that contain a few functions that are used in the tutorial.

Besides the core features of R we will apply the caret package (**Kuhn et al.**, 2008) for machine learning, the t-SNE package (**van der Maaten and Hinton**, 2008) and the ggplot2 package by Hadley Wickham.

1.1 SETUP R ENVIRONMENT

```
> # The file path to the provided input data
> file <- ``dataAllTags.txt''
> # a prefix for all result files created in this tutorial
> basename <- ``allYears``
> # set up environment
> require(ggplot2)
> require(caret)
> require(tsne)
> # allows to reproduce any ``random'' results - remove later!
> set.sedd(123)
> # the file paths to the provided .R tutorial source files
> source(``util.R'')
> source(``svm.R'')
```

1.2 READ AND PREPARE THE INPUT DATA

```
> # read data into dataframe and normalize
> return <- parseTextfile(file)
> dataframe <- data.frame(return[1])
> # Normalize our data
> dataframe <- normalize(dataframe)
> #
> # Get our three factors
> groups.factor.years <- as.factor(return[2][[1]])
> groups.factor.growth <- as.factor(return[3][[1]])
> groups.factor.variety <- as.factor(return[4][[1]])
> #
> # Define prediction factor and partitions
> prediction.factor <- groups.factor.growth
> partitioning <- createDataPartition(
prediction.factor,p=.8,list=FALSE)
> trainAtFactor <- getPartitionFromList(
prediction.factor,partitioning)
> testAtFactor <- getPartitionFromList(
prediction.factor,partitioning,subtract=TRUE)
> trainAtData <- getPartitionFromDataframe(
dataframe,partitioning)
> testAtData <- getPartitionFromDataframe(
dataframe,partitioning,subtract=TRUE)
```

Please note that the here provided code for reading the data is tailored to our input file and will most assuredly not work as such with differently formatted input files.

1.3 PERFORM SVM AND CALCULATE VARIABLE IMPORTANCES

```
> svmResult <- performSVMtraining(
trainAtData,trainAtFactor)
# the confusion matrix contains good measures for our results> svmCM <-
predictionConfusion(
svmResult,testAtData,testAtFactor)
```

```
> varImp <- getImpVars( svmResult )
> # Top ten variable importances
> topVarImp <- rownames(varImp)[1:10]
> # Plot the variable importances
> varImpResult <- varImp( svmResult )
> plot(varImpResult)
> savePlot(
filename=paste(basename,``-varImp.png'',sep=``''),
type=``png``)
```

1.4 CREATE PCA

```
> pca <- createPCA(dataframe,T)
> pcaPlot <- createPCAplot(
pca=pca,
choices=1:2, # choose principal components 1 and 2
chromas=rownames(dataframe),metabolites=colnames(dataframe),
groups.factor.color=groups.factor.variety, # color pca by cultivar
groups.factor.shape=groups.factor.growth) # shapes by farming system
> ggsave(paste(basename,''-pca-1-2.png``,sep=''``),
plot=pcaPlot,width=12,height=12,dpi=72)
```

```
 > # Plot PCA loadings
> pcaLoadingsPlot <- createLoadingsPlot(pca)
> ggsave(paste(basename,''-pcaLoadings.png``,sep='' ``),
plot=pcaLoadingsPlot,width=12,height=12,dpi=72)
```

```
 > # Plot explained variances
> pcaVarPlot <- createVARplot(pca)
> ggsave(paste(basename,''-pcaVariance.png``,sep=''``),
plot=pcaVarPlot,width=12,height=12,dpi=72)
```

1.5 CREATE T-SNE

```
> tSNE <- createTSNE(dataframe, 50, 2, 1000)
> tsne.dataframe <- as.data.frame(tSNE,
row.names=rownames(dataframe))
> tsnePlot <- createTsnePlot(tSNE,
groups.factor.color=groups.factor.growth, # color by farming system
groups.factor.shape=groups.factor.years) # shape by year
> ggsave(paste(basename,''-tsne.png``,sep=''``),
plot=tsnePlot,width=12,height=12,dpi=72)
```

2 SUPPLEMENTARY FIGURES

The below presented variable importance estimations (Fig. 1 - 3) have been calculated on basis of the respective support vector machine (SVM) training results. They were created using R

(**R_Development_Core_Team**, 2011) and the caret package (**Kuhn et al.**, 2008). Caret calculates variable importance in SVMs using the $filterVarImp$ method, applying receiver-operating-characteristics (ROC) curves of each predictor.

REFERENCES

Kuhn, S., Egert, B., Neumann, S., and Steinbeck, C. (2008), Building blocks for automated elucidation of metabolites: machine learning methods for NMR prediction., *BMC bioinformatics*, 9, 400, doi:10.1186/1471-2105-9-400

R_Development_Core_Team (2011), R: A Language and Environment for Statistical Computing, doi: ISBN3-900051-07-0

van der Maaten, L. and Hinton, G. (2008), Visualizing data using t-SNE, *Journal of Machine Learning Research*, 9, 85, 2579–2605

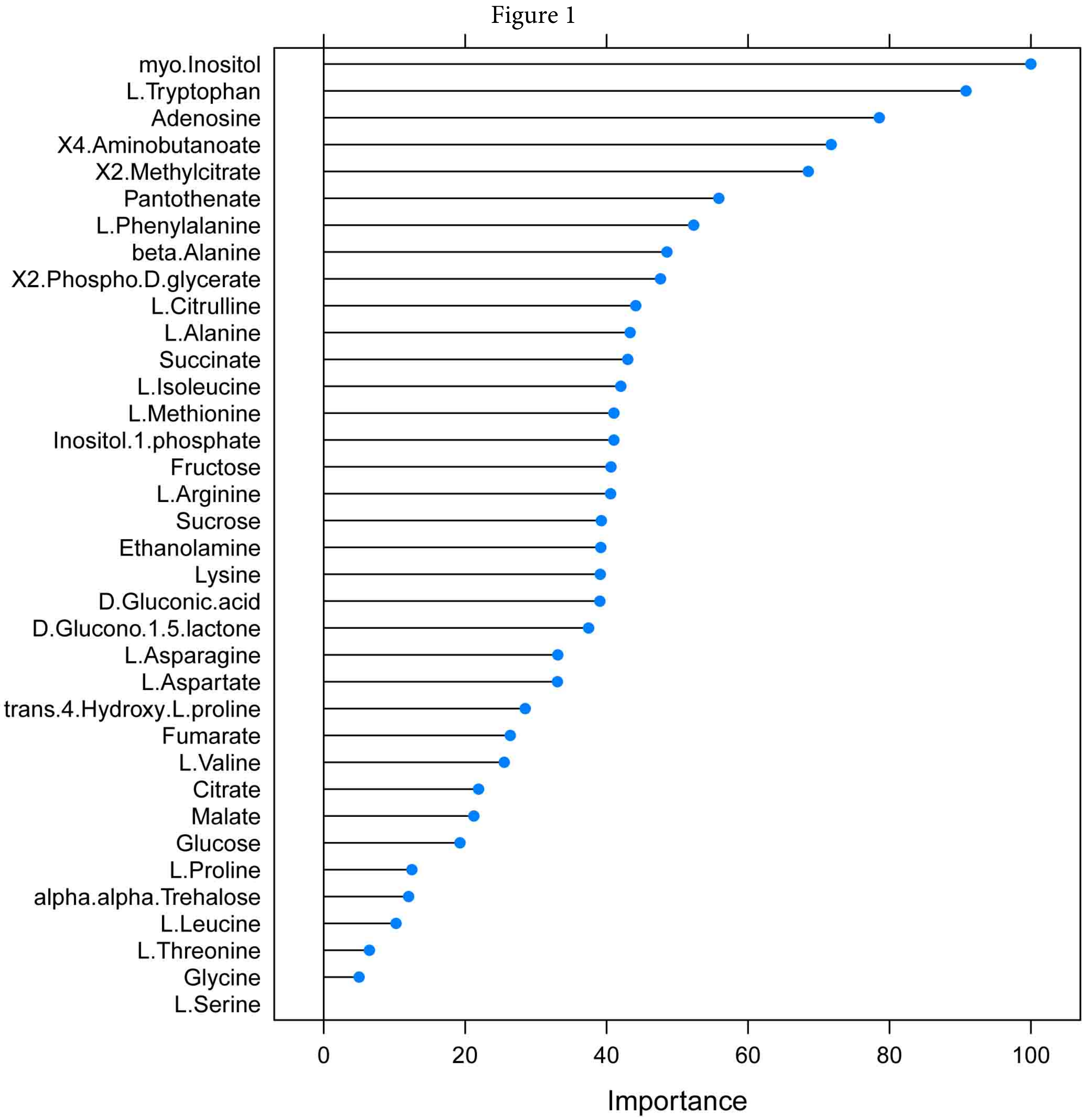

Supplementary Figure 1. Variable importance estimation based on the SVM results for year 2007.

Figure 2

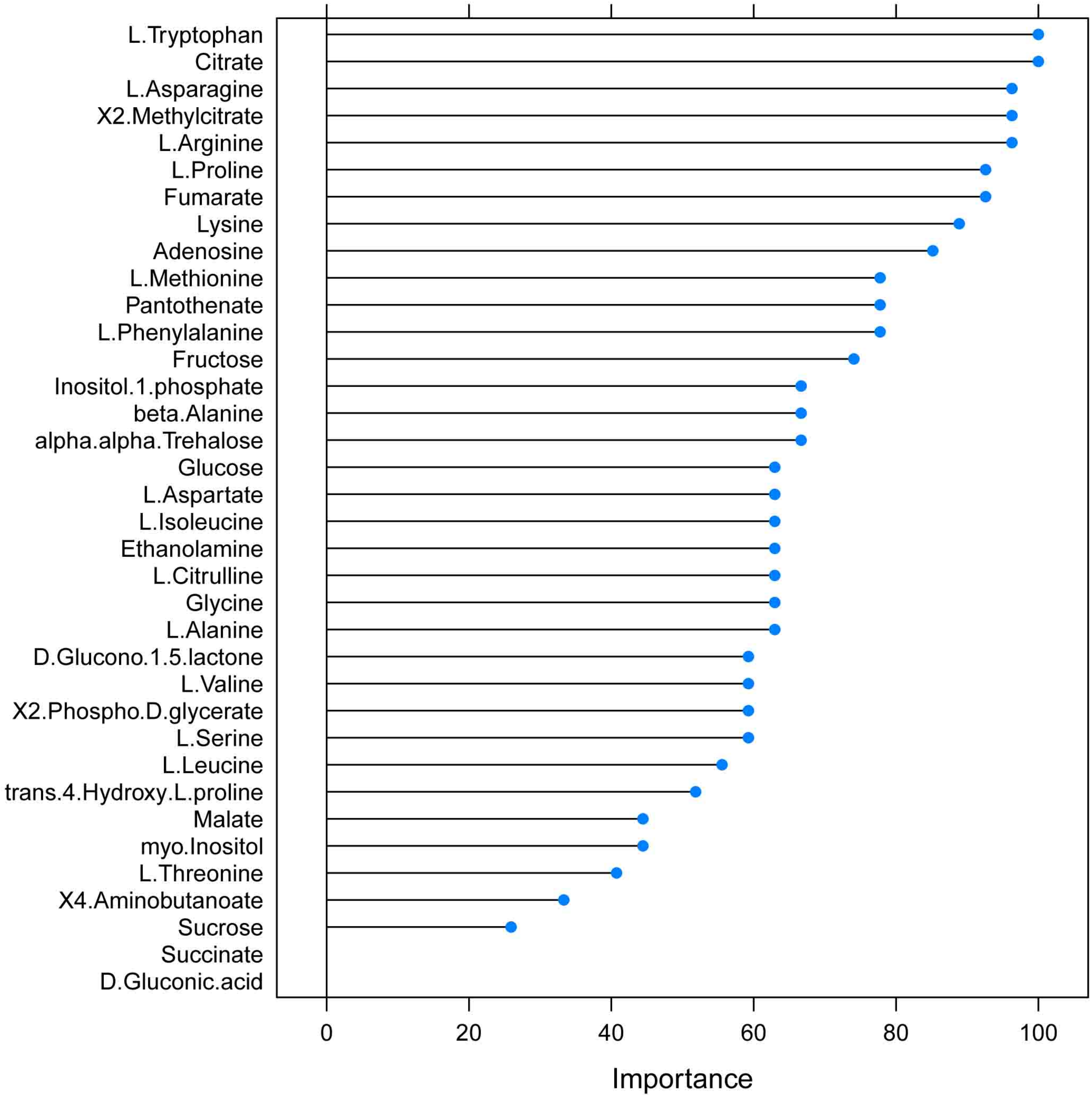

Supplementary Figure 2. Variable importance estimation based on the SVM results for year 2009.

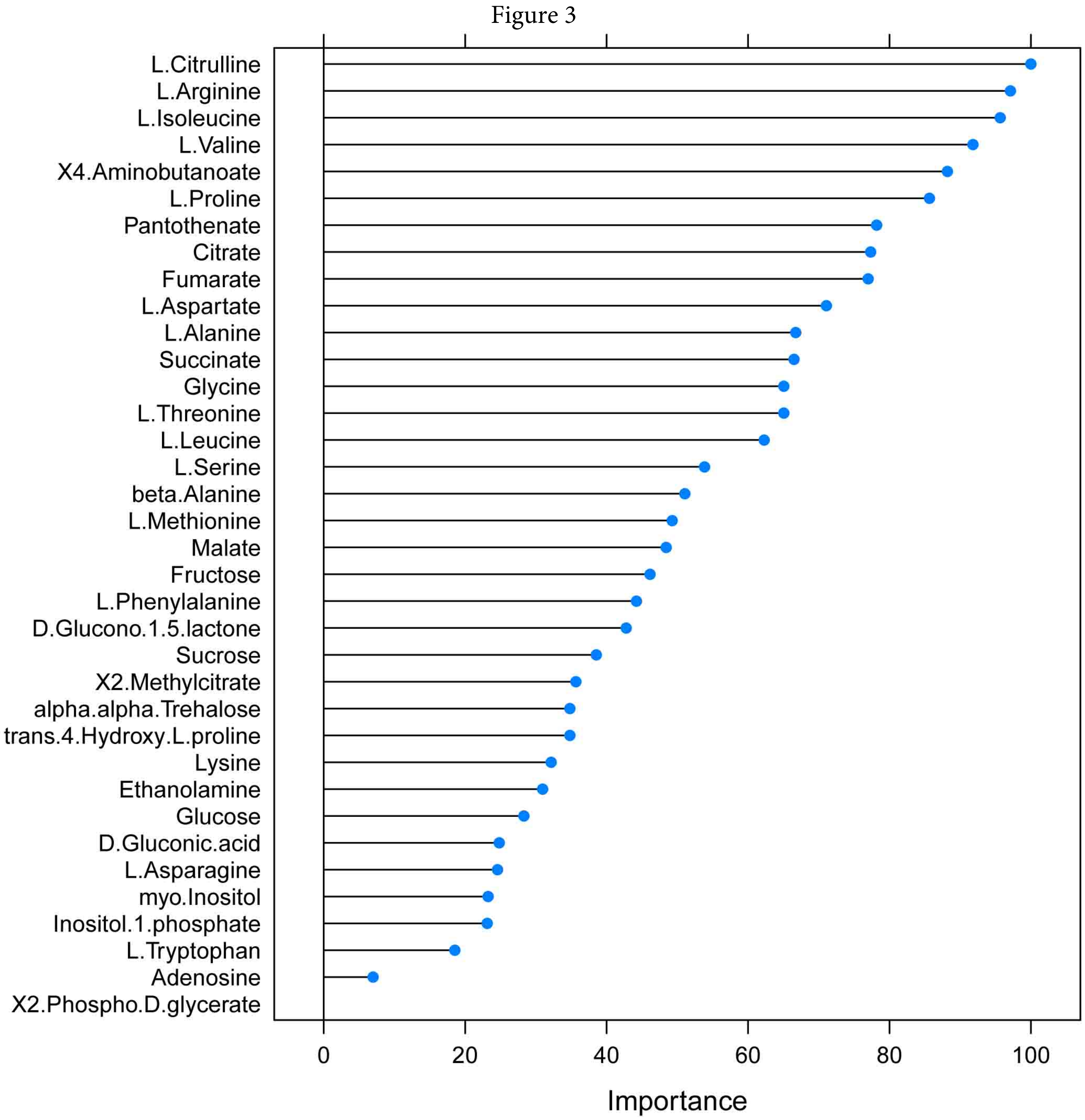

Supplementary Figure 3. Variable importance estimation based on the SVM results for year 2010.
